# Inflammatory bowel disease-specific health-related quality of life instruments: a systematic review of measurement properties

**DOI:** 10.1186/s12955-017-0753-2

**Published:** 2017-09-15

**Authors:** Xin-Lin Chen, Liang-huan Zhong, Yi Wen, Tian-Wen Liu, Xiao-Ying Li, Zheng-Kun Hou, Yue Hu, Chuan-wei Mo, Feng-Bin Liu

**Affiliations:** 1 College of Basic Medical Science, Guangzhou University of Chinese Medicine, Guangzhou, China; 2 Guangzhou University of Chinese Medicine, Guangzhou, China; 3 The First Affiliated Hospital, The First Clinical College, Guangzhou University of Chinese Medicine, Guangzhou, China; 4 Guangdong Province Hospital of Chinese Medicine, Guangzhou University of Chinese Medicine, Guangzhou, China; 5 Jiangmen Wuyi Traditional Chinese Medicine Hospital, Jiangmen City, Guangdong Province China

**Keywords:** Inflammatory bowel disease, Quality of life, Measurement properties, Instrument

## Abstract

**Background:**

This review aims to critically appraise and compare the measurement properties of inflammatory bowel disease (IBD)-specific health-related quality of life instruments.

**Methods:**

Medline, EMBASE and ISI Web of Knowledge were searched from their inception to May 2016. IBD-specific instruments for patients with Crohn’s disease, ulcerative colitis or IBD were enrolled. The basic characteristics and domains of the instruments were collected. The methodological quality of measurement properties and measurement properties of the instruments were assessed.

**Results:**

Fifteen IBD-specific instruments were included, which included twelve instruments for adult IBD patients and three for paediatric IBD patients. All of the instruments were developed in North American and European countries. The following common domains were identified: IBD-related symptoms, physical, emotional and social domain. The methodological quality was satisfactory for content validity; fair in internal consistency, reliability, structural validity, hypotheses testing and criterion validity; and poor in measurement error, cross-cultural validity and responsiveness. For adult IBD patients, the IBDQ-32 and its short version (SIBDQ) had good measurement properties and were the most widely used worldwide. For paediatric IBD patients, the IMPACT-III had good measurement properties and had more translated versions.

**Conclusions:**

Most methodological quality should be promoted, especially measurement error, cross-cultural validity and responsiveness. The IBDQ-32 was the most widely used instrument with good reliability and validity, followed by the SIBDQ and IMPACT-III. Further validation studies are necessary to support the use of other instruments.

**Electronic supplementary material:**

The online version of this article (10.1186/s12955-017-0753-2) contains supplementary material, which is available to authorized users.

## Background

Inflammatory bowel diseases (IBD) are characterized by chronic, uncontrolled and relapsing inflammation of the gastrointestinal tract, which encompasses Crohn’s disease (CD) and ulcerative colitis (UC). Health-related quality of life (HRQoL) is defined as a broad, multidimensional concept comprising patients’ physical health (including disease), psychological state, level of independence, social relationships, personal beliefs and relationship to their environment [1, 2]. The evaluation of HRQoL for patients with IBD in clinical research and clinical practice enhances the understanding of the disease impact and the effects of treatments on the disease. Thus, the evaluation of HRQoL should be recognized as an important outcome indicator by patients and their clinicians.

Up to now, a large number of IBD-specific HRQoL instruments have been developed and validated for the IBD patients [3–7]. These instruments have been used to assess patients’ understanding of IBD symptoms and the subjective perception of the illness in clinical practice and research [3, 4]. They have also been used to compare the effect of treatment strategies and to provide evidence for health policy makers [3–5].

Several researchers have conducted reviews that measure the HRQoL of patients with IBD [3–8]. However, the reviews only enrolled some of the instruments, while other instruments are commonly ignored. The measurement properties and methodological quality of measurement properties should be evaluated systematically for clinical practitioner and researchers. We aimed to comprehensively collect all of the eligible IBD-specific HRQoL instruments to gain an understanding of their measurement properties. Therefore, the aim of this systematic review was to critically appraise and compare the measurement properties of the instruments to help clinicians and researchers select an appropriate instrument.

## Methods

### Inclusion and exclusion criteria

This study was conducted following the guideline of the preferred reporting items for systematic reviews and meta-analysis (PRISMA statement) [9]. Articles were included if they fulfilled the following criteria: (1) Types of patients: Patients diagnosed as CD, UC or IBD were enrolled. Patients with other diseases (infectious colitis, ischemic colitis, irritable bowel syndrome, etc.) were excluded. (2) Types of instruments: The HRQoL instruments developed and validated for patients with CD, UC or IBD were eligible. HRQoL was defined as a broad, multidimensional concept comprising patients’ physical health (including disease), psychological state, level of independence, social relationships, personal beliefs and relationship to their environment. Both the self-administered and rater-administered instruments were included. The instruments for child or adult patients were included. (3) Types of languages: The full-text articles were published in English. General HRQoL instruments were excluded, such as the SF-36. Disease-specific instruments not related or only partially related to IBD were also excluded, such as the gastrointestinal quality of life index [10].

### Literature search

The following relevant electronic databases were searched for English-language articles: Medline (via Pubmed) and EMBASE. The search period was from the inception of the databases to May 31th 2016. The search strategy for Medline (see Additional file 1: Appendix S1) consisted of 3 types of search terms for the following: (1) IBD, UC or CD; (2) HRQoL; and (3) measurement properties. The latter two filters were developed according to the syntax established by Kotecha et al. [11].

In addition, Google Scholar was used to search for relevant articles and literature. The citations of the reviews and the references of included articles were also checked. The patient-reported outcome and quality of life instruments database (website: https://eprovide.mapi-trust.org/) was searched for eligible instruments. Two review authors (XLC, FBL) independently performed the literature search. Disagreements between the two authors were resolved by discussion with another author (LHZ).

### Literature extraction

A set of questions regarding the characteristics of the instruments were drafted. The characteristics were as follows: Which type of disease does the instrument assess (IBD, UC or CD)? How is the instrument administered (self-administered or rater-administered)? How long does it take to complete (completion time)? At what time does it measure the HRQoL of the patients (recall period)? How many items does it contain? What is the form of the item (response options: including Likert or visual analogue scale [VAS])? What is the range of the scores? What domains does it contain? Are classical test theory and item response theory applied? Data about the first author, year of publication, the full and abbreviated names of the instrument and the country of origin (the first version) were also collected.

The methodological quality of measurement properties was assessed according to the consensus-based standards for the selection of health measurement instruments (COSMIN) checklist with a 4-point scale [12–14]. The COSMIN had the following items: internal consistency, reliability (test-retest reliability), measurement error, content validity, structural validity, hypothesis testing, cross-cultural validity, criterion validity and responsiveness. For each instrument, the measurement properties were rated as “poor”, “fair”, “good” or “excellent” based on predefined criteria [12–14]. The definitions of measurement properties for measurement properties based on COSMIN checklist are shown in Additional file 1: Appendix S2. The following measurement properties of the instruments were also evaluated: reliability (internal consistency, test-retest reliability), content validity (interviews/focus groups, pilot test), structural validity (convergent/divergent, discriminant), criterion validity and responsiveness.

The methodological quality of measurement properties was based on the original version, except that cross-cultural validity was based on the translated versions. Two of the three review authors (XLC, LHZ or YW) independently performed the article selection, screened and extracted the characteristics of the instruments and assessed the measurement properties. Disagreements between the two authors were resolved by discussion with another author (TWL or XYL).

## Results

**Fig. 1:**
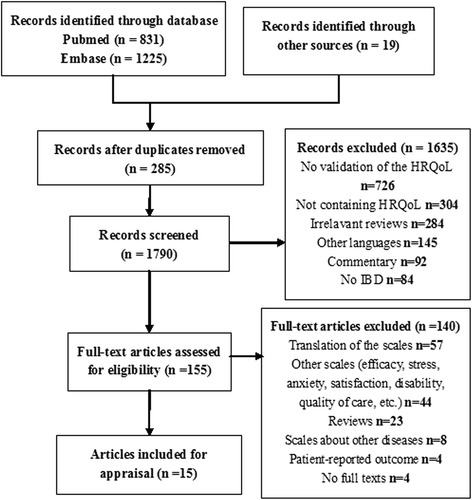
Flow chart of the search strategy

In total, 2075 articles were identified through the search, and 155 potential articles were included for the full text evaluation (Fig. 1). After manually evaluating the full text, 19 IBD-specific HRQoL instruments were identified. The Crohn’s and colitis quality of life questionnaire [15], inflammatory bowel disease impact and symptom scales [16], the Crohn’s disease patient-reported outcomes [17] and ulcerative colitis patient-reported outcomes [18] were excluded due to the lack of full text. At last, 15 articles investigating 15 IBD-specific instruments were included [19–33]. Among them, three instruments were for paediatric IBD patients, and the others were for adult IBD patients.

The basic characteristics of the included instruments are shown in Table 1. The quality-of-life index for pediatric inflammatory bowel disease (IMPACT) [19, 34], IMPACT-II [20, 35] and IMPACT-III [21, 36] were IMPACT series instruments. The IMPACT series instruments were for paediatric IBD patients. The 32-item inflammatory bowel disease questionnaire (IBDQ-32) [22, 37], the 36-item inflammatory bowel disease questionnaire (IBDQ-36) [24], the short inflammatory bowel disease questionnaire (SIBDQ) [23, 38] and the 9-item inflammatory bowel disease questionnaire (IBDQ-9) [25] were IBDQ series instruments. All the instruments were developed for patients with IBD, except the Crohn’s life impact questionnaire (CLIQ) for patients with CD [33]. All of the instruments were developed in North American and European countries. All of the instruments were self-administered. Four instruments also had rater-administered versions [22, 23, 25, 29]. Response options in 9 instruments were Likert scales [21–25, 27, 28, 31, 32], and others were VAS scales.

**Table 1:** Characteristics of the included instruments

Instrument	Author	Year	Full name	Country	Type of disease	Mode of administration	Recall period	Completion time	Number of items	Response options	Number of domains	What scores	Range of scores
For paediatrics													
IMPACT	Griffiths AM [19]	1999	The Quality-of-Life Index for Pediatric Inflammatory Bowel Disease	Canada	PIBD	Self	NA	10 ~ 15 min	33	7 cm VAS	6	Total, domain	0 ~ 231(worst-best)
IMPACT-II	Loonen HJ [20]	2002	The Quality-of-Life Index for Pediatric Inflammatory Bowel Disease II	Netherlands	PIBD	Self	Past 2 weeks	10 ~ 15 min	35	7 cm VAS	6	Total, domain	0 ~ 245(worst-best)
IMPACT-III	Ogden CA [21]	2008	The Quality-of-Life Index for Pediatric Inflammatory Bowel Disease III	UK	PIBD	Self	Past 2 weeks	10 ~ 15 min	35	0–4 Likert	5	Total, domain	0 ~ 100(worst-best)
For adults													
IBDQ-32	Guyatt G [22]	1989	The 32-item Inflammatory Bowel Disease Questionnaire	Canada	IBD	Self, rater	Past 2 weeks	NA	32	1–7 Likert	4	Total, domain	32 ~ 224(worst-best)
SIBDQ*	Irvine EJ [23]	1996	The Short Inflammatory Bowel Disease Questionnaire	Canada	IBD	Self, rater, IVRS	Past 2 weeks	<10 min	10	1–7 Likert	4	Total, domain	10 ~ 70(worst-best)
IBDQ-36	Love JR [24]	1992	The 36-item Inflammatory Bowel Disease Questionnaire	Canada	IBD	Self	Past 2 weeks	NA	36	1–7 Likert	5	Total, domain	1 ~ 7(worst-best)
IBDQ-9*	Alcalá MJ [25]	2004	The 9-item Inflammatory Bowel Disease Questionnaire	Spain	IBD	Self, rater	Past 2 weeks	NA	9	1–7 Likert	1	Total	0 ~ 100(worst-best)
RFIPC	Drossman DA [26]	1991	The Rating Form of IBD Patient Concerns	USA	IBD	Self	Today	NA	25	100-mm VAS	4	Total, domain	0 ~ 100(best-worst)
CCQIBD	Farmer RG [27]	1992	The Cleveland Clinic Questionnaire for Inflammatory Bowel Disease	USA	IBD	Self	Past 2 months	15 ~ 20 min	47	1–5 Likert	4	Total	31 ~ 90(worst-best)
PIBDQL	Martin A [28]	1995	The Padova Inflammatory Bowel Disease Quality of Life	Italy	IBD*	Self	NA	NA	29	0–3 Likert	4	Total, domain	0 ~ 87(best-worst)
CGQL	Fazio VW [29]	1999	The Cleveland Global Quality of Life	USA	IBD*	Self, rater	Today	NA	3	0–10 VAS	3	Total, domain	0 ~ 1(worst-best)
SHS	Hjortswang H [30]	2001	The Short Health Scale	Sweden	IBD	Self	NA	NA	4	100-mm VAS	4	domain	0 ~ 100(best-worst)
EIBDQ	Smith GD [31]	2002	The Edinburgh Inflammatory Bowel Disease Questionnaire	UK	IBD	Self	Past 2 weeks	NA	13	0–1 or 0–3 Likert	3	Domain	NA(NA)
CLIQ	Wilburn J [33]	2015	The Crohn’s Life Impact Questionnaire	UK	CD	NA	NA	NA	36	NA	2	Total, domain	NA(best-worst)
CUCQ	Alrubaiy L [32]	2015	The Crohn’s and ulcerative colitis questionnaire	UK	IBD	Self	Past 2 weeks	<10 min	8	0–3 Likert	1	Total	NA(best-worst)

*CCQIBD* Cleveland Clinic Questionnaire for Inflammatory Bowel Disease (named by the author), *CD* Crohn’s disease, *IBD* inflammatory bowel disease, *IVRS* administration of interactive voice response system, *NA* not available, *PIBD* paediatric inflammatory bowel disease, *Rater* rater-administered, *Self* self-administered, *UC* ulcerative colitis, *VAS* visual analogue scale

IBD*: IBD patients after surgeries, including ileostomy, ileocolonic resection, proctocolectomy and ileoanal anastomosis, restorative proctocolectomy, ileal pouch anal anastomosis; Netherlands*: data from the Netherlands, Norway, Ireland, Portugal, Italy, Greece and Israel;

The SIBDQ* and IBDQ-9* were short versions of the IBDQ-32 and IBDQ-36, respectively

The numbers of domains in the 15 instruments varied from 1 to 6 (Table 2). For the instruments of paediatric IBD, the IMPACT series instruments contained four domains: IBD-related symptoms, physical functioning, emotional functioning and social functioning. For adult IBD patients, some instruments contained the above four domains, whereas some only contained one or two domains. In total, of 55 domains were obtained from all the instruments. (1) Among them, 19 domains were about IBD-related symptoms, which contained bowel or intestinal symptoms (10 domains), systemic symptoms or impairment (6 domains), other symptoms (2 domains) and disease complications (1 domain). (2) Fifteen domains were related to physical functioning or general wellbeing, comprising general quality of life or general wellbeing (5 domains), body image or body stigma (4 domains), functional functioning or impairment (2 domains), energy (2 domains), activity limitations (1 domain) and sexual intimacy (1 domain). (3) Nine domains were about emotional functioning, comprising emotional functioning or impairment (6 domains), disease-related worry (2 domains) and embarrassment (1 domain). (4) Ten domains were about social functioning, containing social functioning (6 domains), social impairment (2 domains) and treatment (2 domains). Another two domains were about information [31] and the total score of the IBDQ-9 (unidimensional) [25].

**Table 2:** Domains of the included instruments

Instrument	IBD-related symptoms (No. of items)	Physical functioning or general wellbeing (No. of items)	Emotional functioning (No. of items)	Social functioning (No. of items)
For paediatrics				
IMPACT	Bowel impairment (6), systemic impairment (2)	Body image (3)	Emotional impairment (11)	Functional/social impairment (11), treatments (3)
IMPACT-II	IBD symptoms (7), systemic symptoms (3)	Body image (3)	Emotional functioning (7)	Social functioning (12), treatment (3)
IMPACT-III	IBD symptoms (5)	Body image (4), energy (4)	Embarrassment (6), worries/concerns about IBD (13)	–
For adults				
IBDQ-32	Bowel symptoms (10), systemic symptoms (5)	–	Emotional functioning (12)	Social functioning (5)
SIBDQ	Bowel symptoms (3), systemic symptoms (2)	–	Emotional functioning (3)	Social functioning (2)
IBDQ-36	Bowel symptoms (8), systemic symptoms (7)	Functional impairment (7)	Emotional functioning (8)	Social impairment (6)
RFIPC	Impact of disease (13), complications of disease (4)	Body stigma (2), sexual intimacy (3)	–	–
CCQIBD	Medical/symptoms (9)	Affect/life in general (11), functional/economic (12)	–	Social/recreational (15)
PIBDQL	Intestinal symptoms (8), systemic symptoms (7)	–	Emotional functioning (9)	Social functioning (5)
CGQL	–	Quality of life (1), quality of health (1), energy level (1)	–	–
SHS	Symptom burden (1)	General wellbeing (1)	Disease-related worry (1)	Social functioning (1)
EIBDQ	Bowel-specific symptoms (6), disease-specific symptoms (5)	–	–	Information (2)*
CLIQ	–	QOL (27), activity limitations (9)	–	–

The IBDQ-9 had only one domain: total score. The CUCQ did not report the domain

Information (2)* in the EIBDQ did not belong to social functioning

-: no domain

The methodological quality of measurement properties based on the COSMIN checklist with 4-point scale ratings is shown in Table 3. All of the instruments were developed and assessed based on classical test theory. Item response theory was also used in the IBDQ-9 and CLIQ. (1) Most of the instruments scored “excellent” or “good” for content validity. The items of these instruments were mainly from interviews with patients, review of the literature and professional experience. The pilot study was used to ensure the applicability of the items in the seven instruments. The domains of these instruments mainly contained IBD-related symptoms, physical, emotional and social functioning (Table 2). For example, the IBDQ-32 contained bowel symptoms, systemic symptoms, emotional and social domains [22]. (2) Most of the instruments scored “good” or “fair” for internal consistency, reliability, structural validity, hypotheses testing and criterion validity. For example, structural validity was rated in 12 instruments. Among them, two instruments scored “excellent” [25, 33], three scored “good” [21, 26, 31], five scored “fair” [19, 20, 22–24] and two scored “poor” [29, 30]. (3) Most of the instruments scored “fair” or “poor” for measurement error, responsiveness and cross-cultural validity. The reasons for responsiveness scoring “fair” or “poor” included: the magnitude of the correlations or differences was not stated; and the criterion for change was not considered as a reasonable gold standard. The reasons for cross-cultural validity scoring “poor” and “fair” included: whether the two translators work independently was not reported; whether the items translated forward and backward was not reported; how differences between the original and translated versions were resolved was not described in detail; the cultural relevance of the translation was not checked; and differential item function between language groups was not assessed.

**Table 3:** COSMIN checklist with 4-point scale ratings of the included instruments

Instrument	Internal consistency	Reliability	Content validity	Measurement error	Structural validity	Hypotheses testing	Criterion validity	Cross-cultural validity	Responsiveness
For paediatrics									
IMPACT	**	***	****	**	**	****	***	NA	NA
IMPACT-II	**	***	****	**	**	***	***	**	NA
IMPACT-III	****	***	****	**	****	***	***	***	NA
For adults									
IBDQ-32	***	***	****	**	**	***	***	****	**
SIBDQ	**	**	***	*	**	***	***	***	***
IBDQ-36	**	NA	**	NA	**	*	NA	NA	NA
IBDQ-9	**	**	***	**	***	****	***	*	****
RFIPC	****	**	****	**	****	**	**	***	*
CCQIBD	NA	*	***	NA	NA	*	**	NA	NA
PIBDQL	NA	NA	**	NA	NA	*	NA	***	NA
CGQL	***	NA	***	NA	*	**	**	**	**
SHS	**	**	**	**	*	***	***	***	***
EIBDQ	***	NA	****	NA	***	**	***	NA	NA
CLIQ	****	***	****	**	***	***	***	NA	NA
CUCQ	**	***	***	**	NA	**	***	NA	****

*Poor, **Fair, ***Good, ****Excellent, NA: not available

The results were based on the original version, except that cross-cultural validity was based on the translated versions

The measurement properties of the instruments are shown in Table 4. (1) The IMPACT series instruments (IMPACT, IMPACT-II and IMPACT-III) were used to assess the HRQoL of paediatric IBD patients. The IMPACT series instruments, especially IMPACT-II and IMPACT-III, had good content validity and were translated into other languages. They were easily administered and contained the main domains (symptoms, physical, emotional and social domains). (2) The IBDQ-32 was considered to be of good measurement properties (content validity) and was proven to be valid, reliable and responsive. The IBDQ-32 contained the main domains: symptom, social and emotional domains. Furthermore, the IBDQ-32 was the most widely used and was translated and back-translated into a variety of languages. (3) The rating form of IBD patient concerns (RFIPC) had good content validity, internal consistency and internal consistency and acceptable responsiveness. Although the original version did not report the responsiveness, its responsiveness was confirmed in the translated version [39]. The RFIPC contained symptoms and emotional domains but did not contain emotional or social domains. (4) The SIBDQ, IBDQ-9, Cleveland global quality of life (CGQL), short health scale (SHS), Edinburgh inflammatory bowel disease questionnaire (EIBDQ) and Crohn’s and ulcerative colitis questionnaire (CUCQ) were short instruments, which were all easily administered and could be completed in a short time. The IBDQ-9, SIBDQ, CUCQ and SHS had good measurement properties. The SIBDQ and IBDQ-9 were short versions of the IBDQ-32 and IBDQ-36, respectively. The SIBDQ was used in the UK, the US, Germany and Spain [40–43]. The SIBDQ contained symptoms, emotional and social domains. The IBDQ-9 was used in Spain and Iran [25, 44], which only contained one domain (total score). The SHS contained symptom burden, general wellbeing, disease-related worry and social functioning. The SHS was used in England, Norway and Sweden [45–47]. The CUCQ was used only in the UK, which should be further evaluated in other languages [32]. (5) For the IBDQ-36, the Cleveland clinic questionnaire for inflammatory bowel disease (CCQIBD) and Padova inflammatory bowel disease quality of life (PIBDQL), limited evidence was available for their measurement properties.

**Table 4:** Measurement properties of the included instruments

	Internal consistency	Test-retest reliability	Interviews/focus groups	Pilot test	Convergent/divergent	Discriminant validity	Measurement error	Criterion validity	Responsiveness
For paediatrics									
IMPACT	0.96	ICC: 0.90	(1) Interview with 82 patients (2) Based on IBDQ (3) Item generation, reduction, and selection procedure	Pilot study, wording of question	Correlation with (1) Current disease activity: −0.54 (2) Colitis symptom score: −0.40 (3) PCDAI: −0.63 (4) Disease activity pattern: −0.43	Higher scores for the patients with quiescent disease (*P* < 0.005)	Test-retest coefficients were calculated.	Correlation with (1) Piers-Harris Happy domain: 0.61 (2) CHQ-87: 0.67	NA
IMPACT-II	0.57 to 0.86	ICC: 0.67 to 0.91	Based on IMPACT	Pilot studies	NA	Higher scores in the patient with severe, moderate symptoms (*P* < 0.05)	Test-retest coefficients were calculated.	Correlations with Tacqol (1) Item: 0.44 to 0.63 (2) Domain: 0.46 to 0.72	NA
IMPACT-III	(1) Factor analysis conducted; (2) 0.74 to 0.88	ICC: 0.66 to 0.84	Based on IMPACT	Pilot study (20 patients)	Paper and computer versions were comparable	Lower scores in the patient with severe, moderate symptom (*P* < 0.05)	Test-retest coefficients were calculated.	Correlations with domain of CHQ-87: 0.47 to 0.72	NA
For adults									
IBDQ-32	0.70	ICC: 0.90 to 0.99	(1) Interview with 97 patients (2) The most frequent and important items	NA	Correlated with CDAI (*r* = −0.67)	Lower scores in patients who required surgery (*P* < 0.05)	Standard deviations of the score changes were of similar magnitude	Correlation of changes in IBDQ and other measures were similar (*P* < 0.05)	Sensitivity to change for the improved or deteriorated patients (*P* < 0.05)
SIBDQ	(1) 0.78 (2) 92% and 90% of the variance in CD and UC were explained	r: 0.65	Based on IBDQ-32	NA	Correlation with (1) SCCAI: −0.42 to −0.85 (2) Seo index: −0.41 to −0.64	Lower scores in the patients with moderate-severe relapse (*P* < 0.05)	Those with unchanged disease status showed no significant difference.	Correlation with the IBDQ-32 (*P* < 0.05)	(1) Sensitivity to change (*P* < 0.05) (2) decreased by −0.93 for the relapsed patients
IBDQ-36	NA	NA	Based on interviewer-administered measure	NA	NA	Lower score for IBD patients than the control (*P* < 0.05)	NA	NA	NA
IBDQ-9	(1) Rasch analysis conducted; (2) UC: 0.95; CD: 0.91	(1) r: 0.76 for UC, 0.86 for CD (2) ICC: 0.82 for UC, 0.84 for CD	Based on IBDQ-36	Pilot test	(1) Item-total correlation: 0.59 to 0.85 (2) Correlation with clinical indices of activity: UC (*r* = 0.70) and CD (r = 0.70)	Lower scores in the patients with moderate-severe relapse (*P* < 0.01)	Scores of the first and second questionnaires correlated significantly	Correlation with IBDQ-36: 0.91	(1) Sensitivity to change (*P* < 0.01) (2) effect size: UC = −2.67, CD = −5.29
RFIPC	(1) Factor analysis conducted; (2) 0.79 to 0.91	(1) r* (instrument): 0.87 (2) r (item): 0.47 to 0.79	(1) 45-min interview (2) items expressed by IBD patients	Add 3 items using pilot study	Associated with greater disease severity, female gender, and lower educational status.	Worse scores for the patients with lower educational status, greater disease severity, female patients and UC patients (*P* < 0.05)	Test-retest coefficients were calculated.	Associated with SCL-90 (*P* < 0.05)	NA
CCQIBD	NA	r: 0.75 to 0.95	(1) Based on other instruments (2) Review of the literature and professional experience	NA	NA	Lower scores for Crohn’s surgical patients (*P* < 0.05)	NA	Associated with sickness impact profile (*P* < 0.05)	NA
PIBDQL	NA	NA	Comprehensive definition of the patients’ health	NA	PIBDQL scores had relationship with daily stools and CDAI score (*P* < 0.05)	(1) Higher scores for the surgical patients (*P* < 0.05) (2) Lower scores for patients than healthy people (*P* < 0.05)	NA	NA	NA
CGQL	0.866	NA	Structured interview	NA	NA	Lower scores in the patients with 0 ~ 5 years after surgery (*P* < 0.05)	NA	Correlation with SF-36: 0.31 to 0.74 (*P* < 0.05)	Sensitivity to change (*P* < 0.001)
SHS	NA	r: 0.71 to 0.91	Theoretic model was presented.	NA	Correlation with PGWB: −0.51 to −0.78	Higher scores for the patients in relapse (*P* < 0.001)	Test-retest coefficients were calculated.	Correlation with (1) IBDQ: −0.41 to −0.78 (2) RFIPC: 0.50 to 0.78	Change in SHS was related with change in disease activity *P* < 0.05)
EIBDQ	(1) Factor analysis conducted (2) Variance extracted: 63% (3) 0.55 to 0.86	NA	Comprehensive review of the IBD literature	Pilot study	Correlation with CDAI (1) CD: 0.52 (2) UC: 0.30	NA	NA	Correlation with SF-36 (1) CD: 0.48 (2) UC: 0.32	NA
CLIQ	0.91–0.93 Rasch analysis conducted, Unidimensional	Reproducibility: 0.91	Literature review, qualitative interviews	Pilot study	Correlation with (1) NHP: 0.53–0.80 (2) U-FIS: 0.79	The QOL in different disease severity patients were significant	Test-retest coefficients were calculated.	Significant differences in CLIQ scores were observed	NA
CUCQ	0.88	ICC: 0.94	Review the literature, consultation with patients and experts	Pilot study (20 patients)	Correlation with (1) HBI: 0.38 (2) SCCAI: 0.35	NA	Test-retest coefficients were calculated.	Correlations with EQ5D (*r* = 0.58), SF-12 (0.63 and 0.65)	(1) Sensitivity to change (*P* < 0.05) (2)Responsiveness ratio: 0.64 (3) standardized response mean: 0.89

*r: correlation coefficients; ICC: intraclass correlation coefficient; NA: not available

CDAI: Crohns disease activity index; CHQ-87: Child Health Questionnaire–Child Form 87; EQ5D: EuroQol 5 dimensions; HBI: Harvey Bradshaw index; NHP: Nottingham Health Profile; PCDAI: paediatric Crohn’s disease activity index; PGWB: psychological general wellbeing; SCCAI: simple clinical colitis activity index; SCL-90: The Symptom Check-List-90; SF-12: The 12-item short-form health survey; SF-36: The 36-item short-form health survey; Tacqol: TNO-AZL Children’s Quality of life questionnaire; U-FIS: Unidimensional Fatigue Impact Scale

The translated versions of the instruments are shown in Table 5. (1) For the instruments of paediatric IBD, the IMPACT-II had 3 translated versions [48–50]. The IMPACT-III had 4 translated versions [51–54]. (2) For the instruments of adult IBD, the IBDQ-32 and RFIPC were the most widely used worldwide. The IBDQ-32 has been translated and validated in 93 languages [55–70] and was found to be reliable and valid in some languages. The IBDQ-32 was also used as an important outcome in randomized controlled trials [71–75]. The RFIPC had at least 6 translated versions [76–82]. The SIBDQ had 4 translated versions [40–43]. The IBDQ-9 [44], IBDQ-36 [83, 84], PIBDQL [85] and CGQL [86] also had translated versions.

**Table 5:** Translated versions of the instruments

Instrument	Translated versions
IMPACT-II	Canadian English [48], US English [49] and Finnish [50]
IMPACT-III	Canadian English [51], US English [52], Croatian [53] and Swedish [54]
IBDQ-32	UK English [55, 56], Dutch [57], Portuguese [58], Greek [59], Swedish [60], Norwegian [61], Japanese [62], German [63], Mandarin [64, 65], Korean [66], Lebanese [67], Brazilian [68], Italian [69] etc.
SIBDQ	UK English [40], German [41], US English [42] and Spanish [43]
IBDQ-36	Spanish [83, 84]
IBDQ-9	Spanish [25], and Iranian [44]
RFIPC	Swedish [76, 77], Norwegian [78], Spanish [79], French [80], Italian [81] and Greek [82]
PIBDQL	English [85]
CGQL	Hindi [86]
SHS	Norwegian [45], English [46] and Swedish [47]

Other instruments did not have translated versions

## Discussion

The present review summarizes an overview of 15 IBD-specific HRQoL instruments with respect to their measurement properties and the methodological quality based on the COSMIN checklist.

According to the results of the COSMIN checklist, most of the instruments did not include all the methodological quality. Only content validity was assessed properly in most of the included instruments. Most of the instruments scored “good” or “fair” for internal consistency, reliability, structural validity, hypotheses testing and criterion validity. The information regarding measurement error, responsiveness and cross-cultural validity was limited or was of poor measurement property because they did not reach the required criteria or because of insufficient information. Our results were consistent with other instruments appraised by the COSMIN criteria, such as irritable bowel syndrome-specific QOL instruments [87]; rheumatoid arthritis-specific QOL instruments [88]; and QOL instruments for infants, children and adolescents with eczema [89]. Most of the IBD-specific instruments did not show adequate methodological quality. One reason for this was that most of the IBD-specific HRQoL instruments were developed before 2010. However, COSMIN guidelines were developed approximately 2010 [12–14]. Therefore, older articles could not follow COSMIN guidelines, and their measurement properties might be underestimated.

Based on the results of the measurement properties and translated versions of the included instruments, some instruments had good psychometric characteristics and were widely used. (1) For paediatric IBD-specific instruments, most of the measurement properties were tested properly, especially the IMPACT-III [21]. The IMPACT-III had the same items as the IMPACT-II. However, The IMPACT-III was on a 0–4 Likert scale, which was easily understood by children. The IMPACT-III was translated into at least 4 translated versions [51–54]. The IMPACT-III was recommended to assess the HRQoL for paediatric IBD patients. (2) For the adult IBD instruments, the IBDQ-32 and SIBDQ (short version of IBDQ-32) had good measurement properties. The two instruments had excellent content validity and proved to be valid, reliable and responsive. The two instruments contained symptoms, emotional and social domains. The two instruments were used widely. The IBDQ-32 has been translated and validated in 93 languages. The SIBDQ was used in the UK, the US, Germany and Spain [40–43]. The IBDQ-9, CGQL, SHS, EIBDQ and CUCQ were all short instruments, which had relatively high methodological quality. However, they had fewer translated versions. The IBDQ-36, CCQIBD, PIBDQL, CGQL and EIBDQ had the lowest measurement properties. The PIBDQL and CGQL instruments were developed and assessed based on IBD patients receiving surgery, and they were translated into other languages. The EIBDQ had not been translated into other languages, which limited its use.

Compared with reviews of IBD-specific instruments published by other authors [3–8], our review had the following advantages. (1) Our review included more eligible IBD-specific HRQoL instruments. For example, the review conducted by Alrubaiy et al. enrolled 10 instruments [8]. Among them, only five instruments were about HRQoL instruments, while others were burden or disability instruments, such as the Crohn’s disease burden questionnaire, the IBD disability score and the IBD disability index. (2) Our review fully evaluated the measurement properties, including content reliability, internal consistency, test-retest reliability, measurement error, convergent/divergent, discriminant validity, criterion validity, cross-cultural validity and responsiveness. Previous reviews did not evaluate criterion validity, discriminant validity or cross-cultural validity for each instrument [8]. Criterion validity and discriminant validity are important features for the instrument. Criterion validity reflects the extent to which scores on a particular instrument relate to a gold standard. Discriminant validity refers to how well the scale can discriminate between different features of the participants.

All of the IBD-specific instruments were developed in North American and European countries. This is likely because the highest incidence and prevalence rates of IBD are in Europe and North America [90]. Another reason might be associated with the popularity of the QOL concepts and the standard procedure for QOL development [91, 92]. In developing countries, researchers mainly focused on translating and back-translating the IBD-specific instruments and used them to assess the QOL of IBD patients.

Although there was a lack of consensus regarding the specific domains among all of the instruments, the common domains measured in the instruments were identified: IBD-related symptoms, physical functioning or general wellbeing, emotional functioning and social functioning. These domains were consistent with the concepts of the common scales, such as the WHOQOL and FACT-G [92–94]. The typical manifestation of IBD included diarrhea with blood, fever, abdominal pain and malnutrition. These symptoms are the most frequently occurring, meaning that the domains contribute the most important information to the IBD-specific instruments.

The limitations of this study were as follows: (1) Non-English articles were not enrolled because of language restrictions; thus, the restriction resulted in limited negative evidence for this study; (2) Articles about the original language were used to assess the measurement properties of the included instruments. The translated articles were not used for the assessment of measurement properties; and (3) Some articles about clinical trials may have been excluded in this review, which resulted in a limited ability to examine responsiveness.

## Conclusions

This review better guides the use of IBD-specific HRQoL instruments and helps clinicians and researchers choose appropriate IBD instruments. The measurement properties scored low for some IBD-specific HRQoL instruments. Based on the characteristics, measurement properties and applications of the instruments, the IBDQ-32 was the most widely used and had the strongest evidence of being reliable, valid and responsive for adult IBD patients. As a short instrument, the SIBDQ also had good measurement properties and was widely used. The IMPACT-III had good measurement properties and was widely used for paediatric IBD patients. For worldwide use of the new instruments, it is necessary to develop instruments according to the standard procedures (for example, the COSMIN) and make sure their measurement properties had excellent or good ratings. New instruments for IBD should take into account IBD-related symptoms and physical, emotional and social domains.

## Additional file

Additional file 1: Appendix S1 and Appendix S2. (DOC 80 kb)

## Abbreviations

CCQIBD: Cleveland clinic questionnaire for inflammatory bowel disease;
CD: Crohn’s disease
CGQL: Cleveland global quality of life
CLIQ: Crohn’s life impact questionnaire
COSMIN: Consensus-based standards for the selection of health measurement instruments
CTT: Classical test theory
CUCQ: Crohn’s and ulcerative colitis questionnaire
EIBDQ: Edinburgh inflammatory bowel disease questionnaire
HRQoL: Health-related quality of life
IBD: Inflammatory bowel diseases
IBDQ-32: 32-item inflammatory bowel disease questionnaire
IBDQ-36: 36-item inflammatory bowel disease questionnaire
IBDQ-9: 9-Item inflammatory bowel disease questionnaire
IMPACT: Quality-of-life index for pediatric inflammatory bowel disease
IMPACT-II: Quality-of-life index for pediatric inflammatory bowel disease II
IMPACT-III: Quality-of-life index for pediatric inflammatory bowel disease III
IRT: Item response theory
PIBDQL: Padova inflammatory bowel disease quality of life
QOL: Quality of life
RFIPC: Rating form of IBD patient concerns
SHS: Short Health Scale
SIBDQ: Short inflammatory bowel disease questionnaire
UC: Ulcerative colitis
VAS: Visual analogue scale
