# Supplementary material for: Inflammatory bowel disease-specific health-related quality of life instruments: a systematic review of measurement properties
Source: Health Qual Life Outcomes. 2017 Sep 15;15:177. doi: 10.1186/s12955-017-0753-2 (PMC5603012; doi:10.1186/s12955-017-0753-2)
Supplement: Additional file 1: — Appendix S1 and Appendix S2. (DOC 80 kb) [file 12955_2017_753_MOESM1_ESM.doc]

## **Appendix S1: Search strategy for Medline**

**1. Condition of interest:**

“inflammatory bowel disease” [MESH] OR “Crohn disease” [MESH] OR “ulcerative colitis” [MESH] OR “inflammatory bowel disease” OR “Crohn's disease” OR “ulcerative colitis”

*AND*

**2. Quality of life measure:**

(HRQL[tiab] OR HRQoL[tiab] OR QoL[tiab] OR quality of life[tw] OR health index*[tiab] OR health indices[tiab] OR ((function[tiab] OR functional[tiab] OR functions[tiab] OR subjective[tiab] OR wellbeing[tiab] OR well being[tiab]) AND (index[tiab] OR indices[tiab] OR **instrument**[tiab] OR instruments[tiab] OR measure[tiab] OR measures[tiab] OR questionnaire[tiab] OR questionnaires[tiab] OR scale[tiab] OR scales[tiab] OR score[tiab] OR scores[tiab] OR survey[tiab] OR surveys[tiab])))

*AND*

**3. Measurement properties:**

("outcome assessment"[MeSH] OR "outcome assessment"[tiab] OR "outcome measure*"[tw] OR "Validation Studies"[pt] OR "psychometrics"[MeSH] OR psychometr*[tiab] OR clinimetr*[tw] OR clinometr*[tw] OR "reproducibility of results"[MeSH] OR reproducib*[tiab] OR "discriminant analysis"[MeSH] OR reliab*[tiab] OR valid*[tiab] OR "coefficient of variation"[tiab] OR coefficient[tiab] OR"internal consistency"[tiab] OR (cronbach*[tiab] AND (alpha[tiab] OR alphas[tiab])) OR (item[tiab] AND (correlation*[tiab] OR selection*[tiab])) OR agreement[tw] OR test-retest[tiab] OR (test[tiab] AND retest[tiab]) OR (reliab*[tiab] AND (test[tiab] OR retest[tiab])) OR stability[tiab] OR interrater[tiab] OR inter-rater[tiab] OR intrarater[tiab] OR intra-rater[tiab] OR intertester[tiab] OR inter-tester[tiab] OR intratester[tiab] OR intra-tester[tiab] OR interobserver[tiab] OR inter-observer[tiab] OR intraobserver[tiab] OR intra-observer[tiab] OR interexaminer[tiab] OR inter-examiner[tiab] OR intraexaminer[tiab] OR intra-examiner[tiab] OR interindividual[tiab] OR inter-individual[tiab] OR intraindividual[tiab] OR intra-individual[tiab] OR interparticipant[tiab] OR inter-participant[tiab] OR intraparticipant[tiab] OR intra-participant[tiab] OR kappa[tiab] OR kappa's[tiab] OR repeatab*[tw] OR ((replicab*[tw] OR repeated[tw]) AND (measure[tw] OR measures[tw] OR findings[tw] OR result[tw] OR results[tw] OR test[tw] OR tests[tw])) OR generaliza*[tiab] OR generalisa*[tiab] OR concordance[tiab] OR (intraclass[tiab] AND correlation*[tiab]) OR discriminative[tiab] OR "known group"[tiab] OR "factor analysis"[tiab] OR "factor analyses"[tiab] OR "factor structure"[tiab] OR "factor structures"[tiab] OR dimension*[tiab] OR subscale*[tiab] OR (multitrait[tiab] AND scaling[tiab] AND (analysis[tiab] OR analyses[tiab])) OR "item discriminant"[tiab] OR "interscale correlation*"[tiab] OR error[tiab] OR errors[tiab] OR "individual variability"[tiab] OR "interval variability"[tiab] OR "rate variability"[tiab] OR (variability[tiab] AND (analysis[tiab] OR values[tiab])) OR (uncertainty[tiab] AND (measurement[tiab] OR measuring[tiab])) OR "standard error of measurement"[tiab] OR sensitiv*[tiab] OR responsive*[tiab] OR (limit[tiab] AND detection[tiab]) OR "minimal detectable concentration"[tiab] OR interpretab*[tiab] OR ((minimal[tiab] OR minimally[tiab] OR clinical[tiab] OR clinically[tiab]) AND (important[tiab] OR significant[tiab] OR detectable[tiab]) AND (change[tiab] OR difference[tiab])) OR (small*[tiab] AND (real[tiab] OR detectable[tiab]) AND (change[tiab] OR difference[tiab])) OR "meaningful change"[tiab] OR "ceiling effect"[tiab] OR "floor effect"[tiab] OR "Item response model"[tiab] OR IRT[tiab] OR Rasch[tiab] OR "Differential item functioning"[tiab] OR DIF[tiab] OR "computer adaptive testing"[tiab] OR "item bank"[tiab] OR "cross-cultural equivalence"[tiab])

*AND*

**4. Limit:**

Filters activated: Publication date to 2016/05/31, Humans

**Appendix S2**

Table 1S. Definitions of the measurement properties based on the COSMIN checklist ([1-3])

|  | Definition |
| --- | --- |
| Internal consistency | The extent to which items in a (sub)scale are inter-correlated, thus measuring the same construct |
| Reliability | The proportion of the total variance in the measurements that is due to ‘true’ differences between patients |
| Content validity | The extent to which the domain of interest is comprehensively sampled by the items in the instrument |
| Measurement error | The systematic and random error of a patient’s score that is not attributed to true changes in the construct to be measured |
| Construct validity | The extent to which scores on a particular instrument relate to other measures in a manner that is consistent with theoretically derived hypotheses concerning the concepts that are being measured |
| Hypotheses testing | The degree to which the scores of an instrument are consistent with the hypotheses |
| Criterion validity | The extent to which scores on a particular instrument relate to a gold standard |
| Cross-cultural validity | The degree to which the performance of the items on a translated or culturally adapted instrument are an adequate reflection of the performance of the items of the original version of the instrument |
| Responsiveness | The ability of an instrument to detect clinically important changes over time |

The criteria for positive ratings are shown in the following references.

1. Terwee CB, Mokkink LB, Knol DL, Ostelo RW, Bouter LM, de Vet HC: **Rating the methodological quality in systematic reviews of studies on measurement properties: a scoring system for the COSMIN checklist.** *Qual Life Res* 2012, **21:**651-657.

2. Mokkink LB, Terwee CB, Patrick DL, Alonso J, Stratford PW, Knol DL, Bouter LM, de Vet HC: **The COSMIN study reached international consensus on taxonomy, terminology, and definitions of measurement properties for health-related patient-reported outcomes.** *J Clin Epidemiol* 2010, **63:**737-745.

3. Mokkink LB, Terwee CB, Patrick DL, Alonso J, Stratford PW, Knol DL, Bouter LM, de Vet HCW: **The COSMIN checklist for assessing the methodological quality of studies on measurement properties of health status measurement instruments: an international Delphi study.** *Quality of Life Research* 2010, **19:**539-549.
